# Targeted Regeneration of Bone in the Osteoporotic Human Femur

**DOI:** 10.1371/journal.pone.0016190

**Published:** 2011-01-14

**Authors:** Kenneth E. S. Poole, Graham M. Treece, Gerard R. Ridgway, Paul M. Mayhew, Jan Borggrefe, Andrew H. Gee

**Affiliations:** 1 Department of Medicine, University of Cambridge, Cambridge, United Kingdom; 2 Department of Engineering, University of Cambridge, Cambridge, United Kingdom; 3 Institute of Neurology, University College London, London, United Kingdom; 4 Department of Diagnostic Radiology, University of Schleswig Holstein, Kiel, Germany; Ohio State University, United States of America

## Abstract

We have recently developed image processing techniques for measuring the cortical thicknesses of skeletal structures in vivo, with resolution surpassing that of the underlying computed tomography system. The resulting thickness maps can be analysed across cohorts by statistical parametric mapping. Applying these methods to the proximal femurs of osteoporotic women, we discover targeted and apparently synergistic effects of pharmaceutical osteoporosis therapy and habitual mechanical load in enhancing bone thickness.

## Introduction

The femur, with its complex load bearing properties and eventual fragility, has been a key anatomical site of study for biologists, engineers and anthropologists across several centuries [1], [2], [3]. Indeed, the seminal concept of ‘functional adaptation’, whereby deformation is sensed by bone cells and transduced into biological signals to optimise skeletal architecture, was based on observations of proximal femoral structure [1], [3], [4]. This idea has long been used to inform orthopaedic practice, and it is in this tradition that we here address an open question in human bone biology: where, precisely, is bone regenerated in response to osteoporosis therapy? With annual hip fracture rates predicted to exceed six million by 2050, and the distribution of cortical bone in the proximal femur believed to be the key determinant of fracture resistance [5], [6], this question is of scientific, social and economic importance. The established clinical intervention to stimulate new bone formation is parathyroid hormone (PTH), administered by daily subcutaneous injection [7]. Invasive animal studies point to a synergistic effect of PTH and localised mechanical stress on bone cells [8], but the more limited experimental techniques applicable to living human beings have, to date, revealed only gross differences in response between heavily loaded sites like the femur and lightly loaded sites like the radius [7].

**Figure 1:**
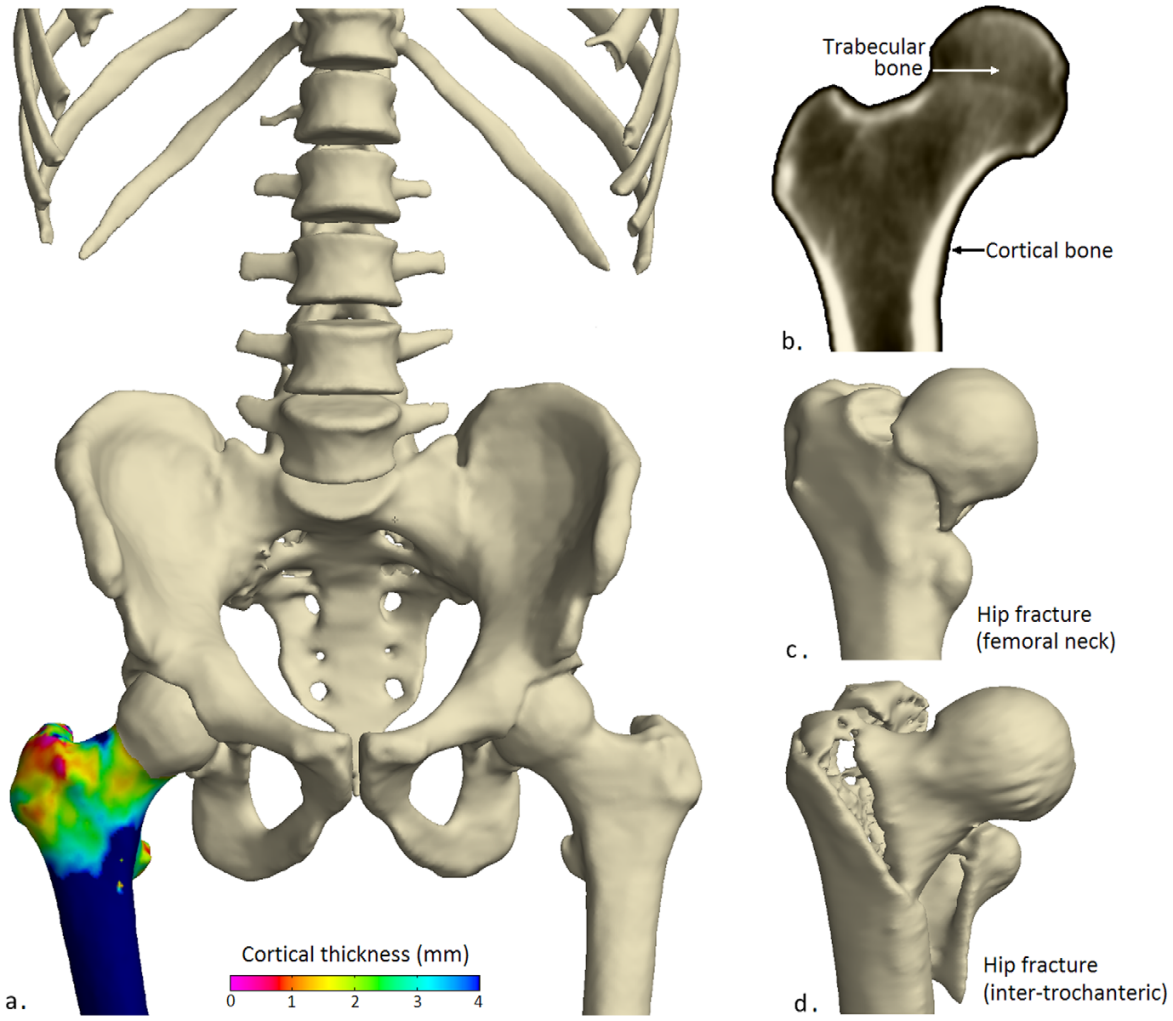
Visualising the femur in health and trauma. (a) A cortical thickness map of a healthy adult male femur with thick (blue/green) cortex at sites of high load during walking, (b) cortical and trabecular bone. Typical fractures in the (c) femoral neck and (d) inter-trochanteric regions.

Further insight has been hampered by the limited resolution of whole-body computed tomography (CT) systems and the perceived futility of using such systems to pinpoint tiny changes in cortical bone distribution. Now, however, a new CT image processing technique [9] allows us to display cortical thickness as a colour map over the bone surface (Fig. 1a), with several thousand independent measurements across each proximal femur and sufficient sensitivity to detect even small changes (∼30 microns) when expressed systematically by a suitably sized cohort. By making reasonable assumptions, that the actual cortical density does not vary dramatically for a given subject at a given time, and that the imaging blur is roughly Gaussian in shape, thickness can be measured to super-resolution accuracy, except at the femoral head where the proximity of the acetabulum is problematic. The methodology has been validated against gold standard thickness measurements obtained from micro-CT scans of cadaveric femurs [9].

## Methods

Here, we use this technique to map the small changes in cortical bone distribution stimulated by pharmaceutical treatment for osteoporosis. In one branch of the EUROFORS study [10], 69 women with severe osteoporosis were treated with recombinant human PTH (hPTH-(1-34)) for 24 months, with quantitative CT scans at baseline and 24 months. All data from this cohort was analysed in the present study, with the exception of those femurs compromised by metalwork and those where one or both of the CT scans did not extend as far as the lesser trochanter. This yielded 119 femurs from 65 women (mean age 67.5±6.8 years). Participants were from twelve investigative centres in Germany and Spain [11]. Institutional Review Board approval was obtained from each of the clinical study sites, and written informed consent for EUROFORS (ClinicalTrials.gov ID NCT00191425) and the QCT sub-study were obtained from each participant. The present analysis was approved by Cambridgeshire 4 research ethics committee (LREC07/H0305/61).

Analysis of the 238 thickness maps followed established practice within the neuroimaging community, who have pioneered techniques for statistical inference from dense, spatially correlated data. To account for variations in inter-subject morphology, each map was spatially realigned with a canonical femur surface using a B-spline free-form deformation [12] calculated by the iterative closest point registration algorithm [13]. The spatially normalized maps were then smoothed with a 10 mm full-width-half-maximum filter. Thickness changes were summarised for each subject by subtracting the baseline maps from the 24-month maps and averaging the left and right responses where both were available. This resulted in 65 summarised response maps, 54 derived from averaged left-right data and 11 from single femurs. Formal inference was accomplished by statistical parametric mapping [14], as implemented in the SurfStat package [15]. The model fitted to the summarised data comprised a constant term and a random-effect term for left-right averaging: this allows for unequal variances between the averaged and unaveraged data. T-statistics were calculated to test the significance of the constant term, corresponding to positive thickness change. Random field theory then furnished *P*-values, corrected for multiple comparisons to control the overall image-wise chance of false positives.

## Results and Discussion

**Figure 2:**
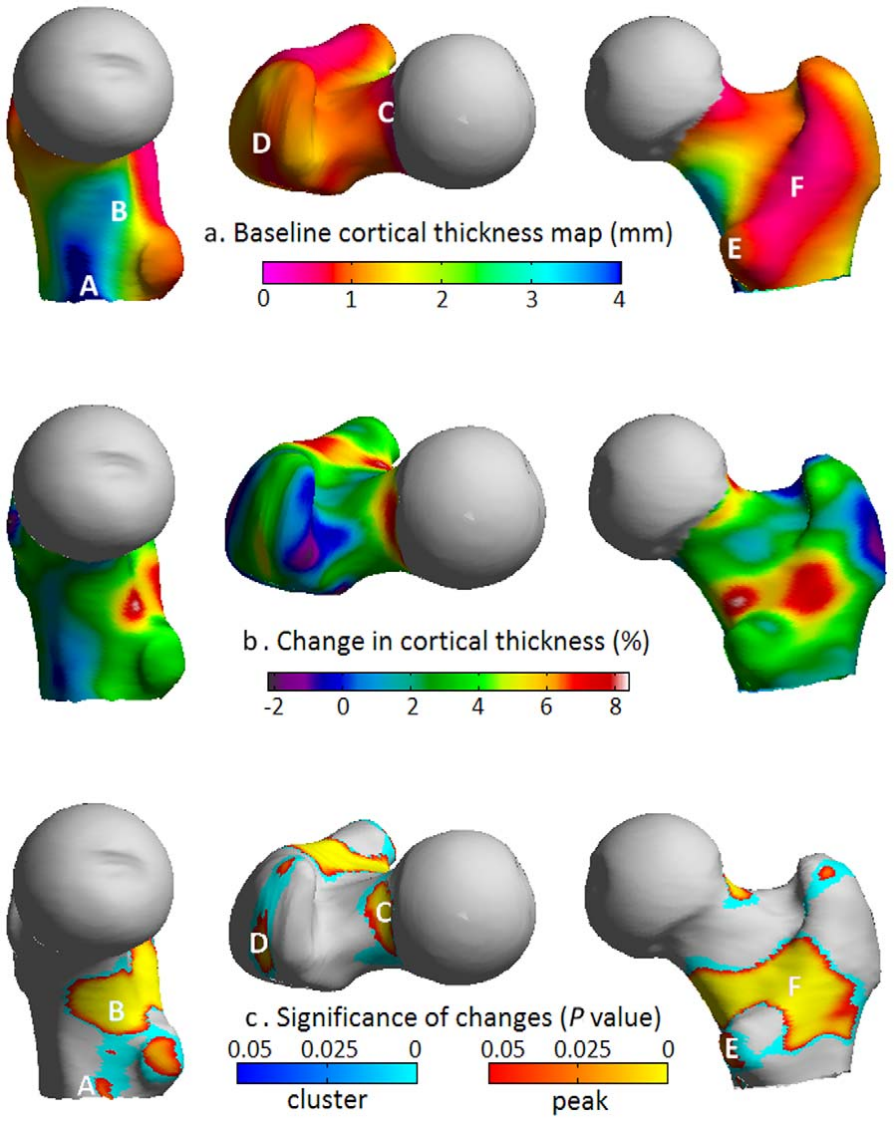
Cortical thickness maps showing severe osteoporosis and the increase in thickness following hPTH-(1-34) treatment. (a) Medial, superior and posterior views of the average pre-treatment cortical thickness map in advanced osteoporosis showing remnants of thicker, highly loaded bone (blue/green) at A) the inferomedial cortex and B) the calcar femorale regions. Elsewhere there is thin, sub-millimetre cortex (pink/red) at key fracture sites including C) the subcapital superior neck region. Also labelled are the insertion sites of key muscles of locomotion; D) gluteus medius, E) psoas major and F) quadratus femoris (on the inter-trochanteric crest). (b) Post-treatment percentage change and (c) statistical significance maps which together indicate regeneration of bone at A), B), C), D), E) and F).

Baseline measurements reveal the anatomical distribution of cortical thickness seen in advanced osteoporosis (Fig. 2a). The nonuniformly thin cortex is of clinical relevance, since fractures commonly traverse the neck (Fig. 1c) or split the trochanteric region during sideways falls (Fig. 1d) [5]. The variation in thickness partly reflects femoral growth, with thick bone of the inferomedial aspect preserved into late adulthood by lifelong functional adaptation to stress, and a thin cortex apparent superiorly. In other areas, such as the trochanters and their connecting crest, thin cortices overlie predominantly trabecular bone near bony sites of muscle and tendon insertion (entheses). Following hPTH-(1-34) treatment, and in striking contrast to the expected ageing effects [5], the percentage change map (Fig. 2b) shows marked cortical thickening. Figure 2c shows corrected *P*-maps for positive thickness change, based on the magnitude of peaks (sensitive to focal effects) and on the extent of connected clusters exceeding an uncorrected *P*-value threshold of 0.001 (sensitive to distributed effects).

New bone is targeted to regions that predictably encounter high stress during normal locomotion, namely the infero-medial junction of the cortex with the load-bearing calcar femorale, and the head-neck junction of the superior cortex. Both these sites are commonly involved in fracture. No regions of statistically significant thinning are apparent. Foci of new bone are also evident at the entheses of powerful locomotion muscles; on the greater and lesser trochanters at the attachment sites of the gluteus medius and psoas, and at the inter-trochanteric insertion of the quadratus femoris.

Parathyroid hormone is an established clinical intervention to stimulate new bone formation in human osteoporosis. Previous studies have shown that hPTH-(1-34) treatment in postmenopausal women with osteoporosis increases cortical thickness at various skeletal sites, but this is the first to demonstrate the precise regional effects at one of the key sites of fragility fracture, the human hip. hPTH-(1-34) is also known to increase porosity (at least early in treatment) and reduce the mineral density, since new bone stimulated by the drug is relatively under-mineralised compared to older bone [16]. The cortical thickness measurement is robust to any consequent changes to the average cortical density, since the average density value is estimated independently in each data set. It is equally robust to any regional changes in the quality of the CT data, since the extent of the imaging blur is estimated at every measurement location.

Intriguingly, both load and PTH act rapidly on the entombed 'load sensing' osteocytes to profoundly reduce secretion of a key molecular inhibitor of bone formation, sclerostin [17]. Released from inhibition, the osteoblasts (surface targets of sclerostin) can then secrete new bone matrix. Fig. 2b suggests a possible synergistic effect of habitual load and PTH in the human proximal femur, since peak effects are seen at sites that are highly stressed by walking. In the light of this, we ask whether hPTH-(1-34) treatment for osteoporosis might be more effective if combined with enhanced skeletal loading. Cortical thickness mapping is the ideal tool to answer this and other open questions in bone biology.

## References

[1] Roux W (1885). Beitrage zur Morphologie der funktionnellen Anspassung.. Arch Anat Physiol Anat Abt.

[2] Lovejoy CO, Suwa G, Spurlock L, Asfaw B, White TD (2009). The pelvis and femur of Ardipithecus ramidus: the emergence of upright walking.. Science.

[3] Rubin C, Turner AS, Bain S, Mallinckrodt C, McLeod K (2001). Anabolism. Low mechanical signals strengthen long bones.. Nature.

[4] Huiskes R, Ruimerman R, van Lenthe GH, Janssen JD (2000). Effects of mechanical forces on maintenance and adaptation of form in trabecular bone.. Nature.

[5] Mayhew PM, Thomas CD, Clement JG, Loveridge N, Beck TJ (2005). Relation between age, femoral neck cortical stability, and hip fracture risk.. Lancet.

[6] Burr DB (2010). Cortical bone: a target for fracture prevention?. Lancet.

[7] Neer RM, Arnaud CD, Zanchetta JR, Prince R, Gaich GA (2001). Effect of parathyroid hormone (1-34) on fractures and bone mineral density in postmenopausal women with osteoporosis.. N Engl J Med.

[8] Roberts MD, Santner TJ, Hart RT (2009). Local bone formation due to combined mechanical loading and intermittent hPTH-(1-34) treatment and its correlation to mechanical signal distributions.. J Biomech.

[9] Treece GM, Gee AH, Mayhew PM, Poole KE (2010). High resolution cortical bone thickness measurement from clinical CT data.. Med Image Anal.

[10] Eastell R, Nickelsen T, Marin F, Barker C, Hadji P (2009). Sequential treatment of severe postmenopausal osteoporosis after teriparatide: final results of the randomized, controlled European Study of Forsteo (EUROFORS).. J Bone Miner Res.

[11] Borggrefe J, Graeff C, Nickelsen TN, Marin F, Gluer CC (2009). Quantitative Computed Tomography Assessment of the Effects of 24 months of Teriparatide Treatment on 3-D Femoral Neck Bone Distribution, Geometry and Bone Strength: Results from the EUROFORS Study.. J Bone Miner Res.

[12] Rueckert D, Frangi AF, Schnabel JA (2003). Automatic construction of 3-D statistical deformation models of the brain using nonrigid registration.. IEEE Trans Med Imaging.

[13] Besl PJ, McKay ND (1992). A Method for Registration of 3-D Shapes.. IEEE Trans Pattern Anal Mach Intell.

[14] Friston KJ, Holmes AP, Worsley KJ, Poline J-P, Frith CD (1994). Statistical parametric maps in functional imaging: A general linear approach.. Human Brain Mapping.

[15] Worsley K, Taylor J, Carbonell F, Chung M, Duerden E (2009). SurfStat: A Matlab toolbox for the statistical analysis of univariate and multivariate surface and volumetric data using linear mixed effects models and random field theory.. NeuroImage Organization for Human Brain Mapping 2009 Annual Meeting.

[16] Misof BM, Roschger P, Cosman F, Kurland ES, Tesch W (2003). Effects of intermittent parathyroid hormone administration on bone mineralization density in iliac crest biopsies from patients with osteoporosis: a paired study before and after treatment.. J Clin Endocrinol Metab.

[17] O'Brien CA, Plotkin LI, Galli C, Goellner JG, Gortazar AR (2008). Control of Bone Mass and Remodeling by PTH Receptor Signaling in Osteocytes.. PLoS ONE.

